# Chipless RFID Sensor for Measuring Time-Varying Electric Fields Using a Contactless Air-Filled Substrate-Integrated Waveguide Resonator

**DOI:** 10.3390/s24154928

**Published:** 2024-07-30

**Authors:** Amirmasoud Amirkabiri, Dawn Idoko, Behzad Kordi, Greg E. Bridges

**Affiliations:** Department of Electrical and Computer Engineering, Price Faculty of Engineering, University of Manitoba, Winnipeg, MB R3T 5V6, Canada; amirkaba@myumanitoba.ca (A.A.); idokod@myumanitoba.ca (D.I.); gregory.bridges@umanitoba.ca (G.E.B.)

**Keywords:** cavity resonator, chipless RFID, electric field measurement, electromagnetic band gap, substrate-integrated waveguide, tunable resonator

## Abstract

This paper presents a wireless chipless resonator-based sensor for measuring the absolute value of an external time-varying electric field. The sensor is developed using contactless air-filled substrate-integrated waveguide (CLAF-SIW) technology. The sensor employs a low-impedance electromagnetic band gap structure to confine the electric field within the sensor’s air cavity. The air cavity is loaded with varactor diodes whose reverse bias voltage is modified by the to-be-measured external electric field. Variation in the external electric field results in a variation of the sensor’s resonant frequency. The CLAF-SIW sensor offers a high unloaded quality factor, which is required for a long-distance ringback-based interrogation system. A prototype of the proposed sensor is fabricated and tested. It can measure a time-varying external electric field up to 6.9 kV/m, has a sensitivity of 1.86 (kHz)/(V/m), and can be interrogated from a distance of 80 cm. The feasible maximum bandwidth of the external electric field is 25 kHz. The proposed sensor offers a compact planar multilayer structure that can easily be incorporated with a planar antenna and its size can be reduced by selecting a higher operating frequency without an increase in dielectric loss.

## 1. Introduction

The numerous advantages of chipless RFID sensors, which include being passive, capable of wireless interrogation, and fairly easy to fabricate, make them a preferred choice in many sensing and monitoring applications [1,2,3]. A passive sensor does not require a battery or other power source, which facilitates low-cost fabrication, very long-term operation without maintenance, and enables remote sensing in harsh or hazardous environments. These applications include temperature/pressure sensing [4,5,6], pH sensing with temperature compensation [7], food quality monitoring applications [8], relative humidity sensing [9,10], and structural health monitoring applications [11].

Many chipless sensors operate using a resonator-based approach where a change in the resonant frequency of the structure in response to a change in a desired measurand is monitored. The measurand may be an external electric field [12,13], temperature [14], or environmental pressure [15]. Resonator-based passive wireless sensors have been employed in many industrial [16,17,18,19] and medical applications [20,21,22]. The wireless nature of these sensors makes them suited to deployment in settings where physical human presence is impractical, such as high-voltage applications [23,24].

Most existing passive wireless sensors are designed to detect slowly varying measurands, such as humidity, pressure, or temperature, and are incapable of recording signals with fast time-varying changes. To tackle this constraint, a frequency counting approach that can detect millisecond changes has been developed [25]. The use of pulsed radio frequency waves to excite the resonator, followed by the capture of ringback signals, was employed in [12] to measure AC electric fields. This technique is capable of detecting time-varying signals as fast as a few microseconds and is particularly effective for monitoring 60 Hz AC waveforms and the resultant harmonics in power systems.

Passive sensors based on coupled inductive-capacitive (LC) resonators, implemented on a printed circuit board (PCB), have been reported in the literature, e.g., [15,26]. Due to their dependence on near-field coupling, these LC resonator-based sensors have a limited measuring range. Microwave resonator-based sensors that also employ PCB technology have the potential to make far-field measurements [27]. However, many of these sensors include microstrip patch resonators, which are lossy and hence diminish their measuring range [28,29], as the feasibility of far-field remote interrogation using resonator-based wireless sensors strongly relies on the sensor having a high quality factor. This is especially crucial for ringback interrogation techniques. A non-planar hollow metallic waveguide is ideal as a high-quality resonator, but it has a large profile and does not facilitate easy integration with planar components.

Substrate-integrated waveguide (SIW) technology was introduced as a viable planar structure alternative to an all-metallic waveguide [30]. SIW is fabricated using metal sandwiching a dielectric substrate and conducting vias as a replacement for the metallic walls in conventional waveguides. To reduce loss in the overall structure an approach, where the the dielectric is replaced by air, is air-filled SIW (AF-SIW) implemented in multilayer PCB technology [31]. However, it needs a flawless connection between the metallic layers and a lack of air gaps between them, making its fabrication complicated [32,33].

Bayat-Makou and Kishk [34] proposed a contactless air-filled substrate-integrated waveguide (CLAF-SIW) technology to construct AF-SIW structures, in which metalized conducting vias are replaced with a mushroom-shaped electromagnetic band gap (EBG) structure. Using mushroom cells in the EBG structure eliminates the need for physical contact between layers, making CLAF-SIW an appropriate option for multilayered SIW systems [34]. In addition, CLAF-SIW-based technology has the highest unloaded quality factor compared with many other types of multilayer PCB structures [35]. The concept of CLAF-SIW has also been employed in applications such as cavity-backed slot antenna array and phase shifters [36,37].

**Figure 1:**
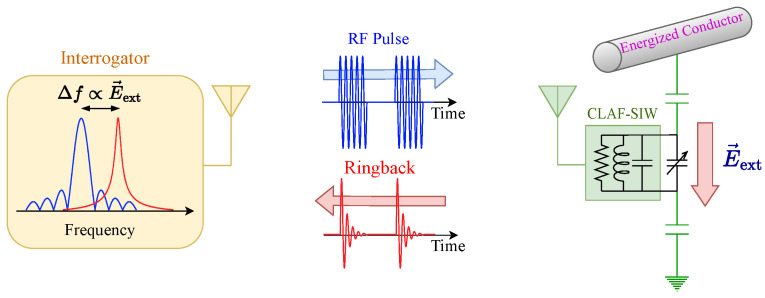
Schematic of the operation of the passive wireless resonator-based electric field sensor. The interrogator transmits RF pulses and receives backscattered ringback signals from the sensor. Variations in the ringback frequency are proportional to the external electric field ${\vec{E}}_{\mathrm{ext}}$.

This paper presents the design, fabrication, and implementation of a novel passive wireless sensor for measuring time-varying electric fields. The sensor consists of an RF resonator loaded with back-to-back varactor diodes whose capacitance changes due to the induced voltage by an external electric field across their terminals, resulting in a change in the resonant frequency of the sensor. The sensor can be interrogated wirelessly by sending and receiving RF pulses at a frequency close to its resonant frequency (see Figure 1). The proposed sensor (and the interrogation system) differs from other resonator-based sensors in that it can measure fast time-varying signals. The high quality factor of the proposed sensor, through the use of a CLAF-SIW resonator, enables reduction of clutter interference and provides longer interrogation distances than existing passive wireless electric field sensors.

## 2. Sensor Design and Fabrication

**Figure 2:**
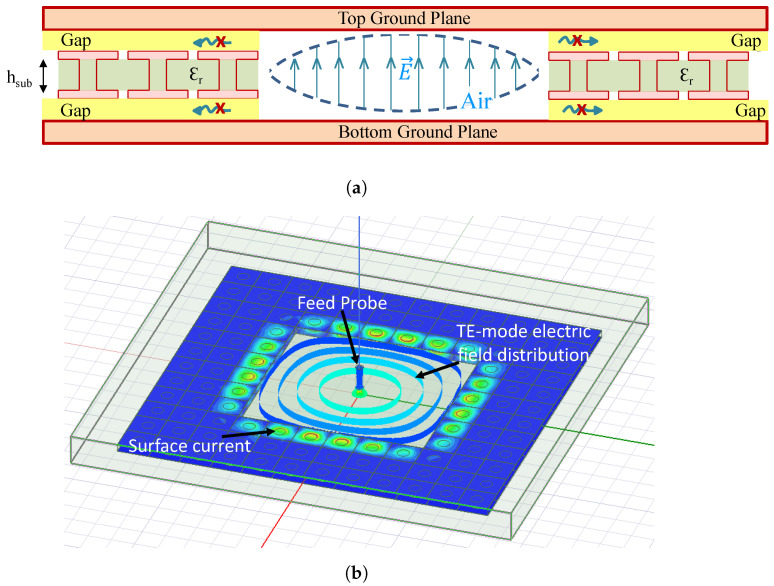
(**a**) A cross-sectional view of the contactless air-filled substrate integrated waveguide resonator. The cavity of the resonator is filled with air and a thin layer of PVC material (labeled as Gap in this figure) isolates the EBG structure from the top and bottom ground planes. (**b**) Simulation of the CLAF-SIW resonator showing electric field and surface current distribution of the dominant TE mode (adapted from [14]).

Traditional substrate-integrated waveguide (SIW) technology has been used to implement numerous sensor applications, e.g., [14,31,38]. The drawback of the SIW technology is the loss in the dielectric-filled substrate, as well as the conducting vias and metalized surfaces. To reduce loss, it has been proposed that the dielectric in the resonator cavity region be replaced with air [32,33]. In [6], it was demonstrated that replacing the low-loss dielectric with air significantly improves the quality factor of a resonator. This technique requires a non-standard multilayer fabrication process with fabrication complexities that can be mitigated by the use of CLAF-SIW technology [34]. In CLAF-SIW, the metalized vias are replaced with an electromagnetic band gap (EBG) structure, often implemented using mushroom-cell patches, as shown in Figure 2a. In this design approach, the electric field is confined to the air cavity region of the resonator (see Figure 2b), minimizing dielectric loss as compared with traditional SIW.

**Figure 3:**
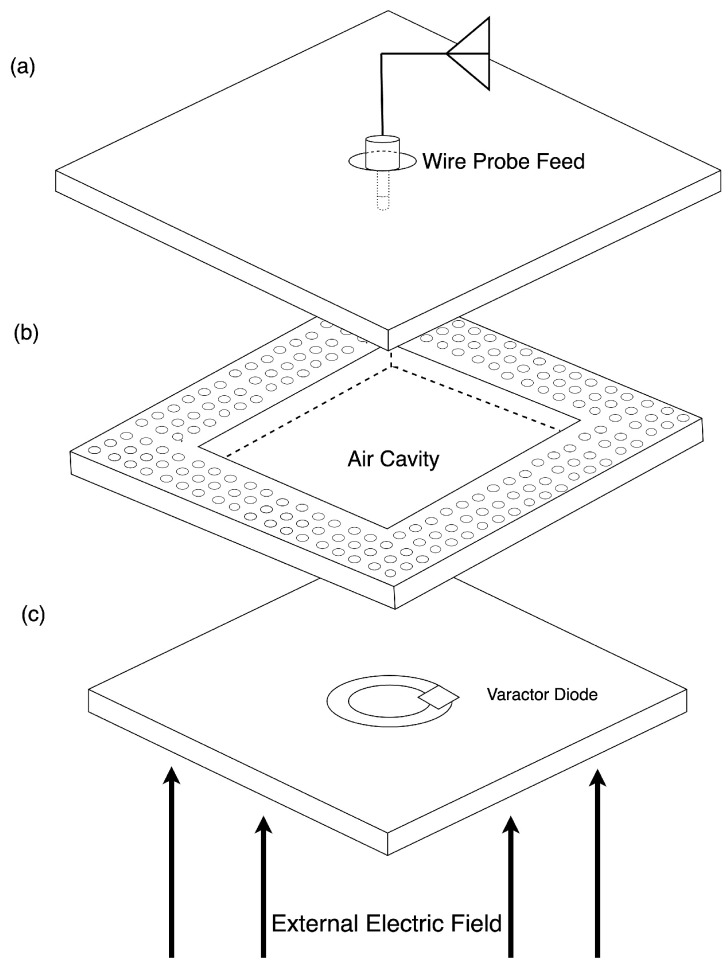
Schematic of the multilayer design of the sensor consisting of the (**a**) top cavity ground plane and the wire probe feed coupled to an external antenna, (**b**) air cavity and the surrounding mushroom cell EBG-type boundary structure, and (**c**) bottom cavity ground plane and the isolated sensing patch for external electric field sensing. This layer has copper cladding on one side. There are thin PVC isolation films between the layers.

Figure 3 shows the concept diagram of the wireless CLAF-SIW electric field sensor. A double-sided copper-clad substrate is the top layer of the cavity. The structure can be fabricated using two-sided PCB technology and then assembled by simply stacking and compressing the layers with a thin PCV film between them. An external antenna connected to a wire probe feed at the center is used to couple to and energize the CLAF-SIW air cavity resonator. The intermediate layer is the air cavity surrounded by the EBG-type mushroom-cell patches, which replace conducting vias. A one-sided copper-clad substrate is used for the bottom conducting plane and contains an isolated electric field sensing patch. An external electric field induces a potential difference across the gap region of the patch. The potential difference biases back-to-back varactor diodes placed across the gap, causing an electric-field dependant capacitive loading on the cavity. This subsequently changes the resonant frequency of the cavity in response to the amplitude change in the external electric field.

**Figure 4:**
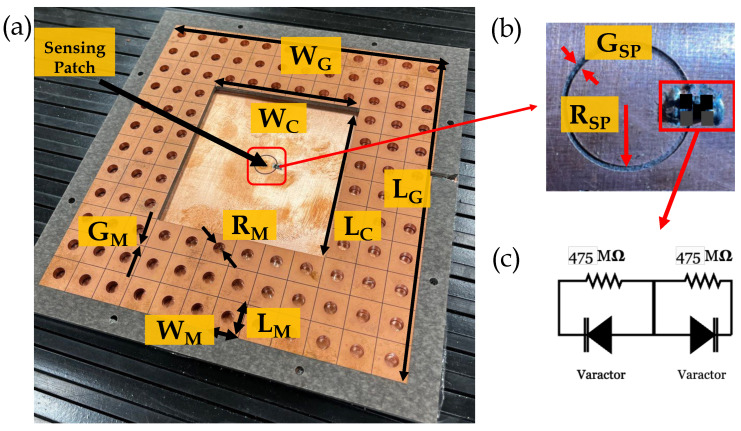
Photograph of the fabricated CLAF-SIW resonator-based sensor showing (**a**) the intermediate layer of the CLAF-SIW resonator and the bottom conducting ground plane where the electric field sensing isolated patch is located. The top ground plane with an SMA probe feed is not shown in this photo, (**b**) circular electric field sensing patch where varactor diodes and resistors are placed across the gap, and (**c**) 475 MΩ resistors in parallel with back-to-back varactor diodes to provide a discharge path for the varactor diodes.

Figure 4 shows the fabricated CLAF-SIW resonator-based electric field sensor. An IsoClad 917 Rogers substrate with a dielectric constant of 2.2 and loss tangent of 0.0013 (at 10 GHz) is used as the CLAF-SIW cavity layer. The substrate thickness and the copper cladding thickness are 3.18 mm and 35 μm, respectively. The cavity layer dimensions given in Table 1 were used to attain a resonant frequency in the 2.4–2.5 GHz ISM band and to prevent degenerate modes. Considering the size of the air cavity, the size of the sensing patch, and the circumference of the ring slot between the sensing patch and the ground plane, the resonant frequency of the CLAF-SIW air cavity is well below the resonant frequency of the isolated sensing patch. In the frequency range of this work, the sensing patch acts as a capacitive load for the air cavity resonant mode. The size of the mushroom-cell patches is central to designing the frequency range of the stop-band in the EBG structure [34,39]. In [14], the dispersion diagram in all transverse directions of the EBG unit cell is presented. The gap between neighboring mushroom cells functions as a capacitance, whereas the metalized via acts as an inductance. Even though the EBG structure provides a low impedance surface at the boundaries of the air-filled region, the resonant frequency is slightly lower than that of a solid-wall or via-based air-cavity resonator [13,40]. The effective length and width of the cavity are larger than the air-cavity region dimensions due to electric field leakage into the first few mushroom-cell patch layers, as shown in Figure 2b. Nevertheless, the electric field is contained primarily within the air cavity, decreasing dielectric loss and improving the quality factor of the structure. Figure 2b shows the electric field distribution of the dominant TE mode where the magnitude of the electric field is the strongest at the center of the air cavity resonator. Placing the sensing patch at the center of the cavity provides the largest coupling between the sensing patch and the CLAF-SIW resonator. A low-loss PVC layer with a thickness of 12.5 μm, relative permittivity of 2.70, and loss tangent of 0.007 is used to maintain a constant gap between the ground planes and the intermediate cavity layer that contains the mushroom cell structures. Details of the top layer and probe feed dimensions are provided in [14,39].

To measure both positive and negative polarities of an external time-varying electric field, two similar varactor diodes are connected back-to-back. For each polarity of the external field, one of the varactor diodes will be in reverse bias while the other varactor will be conducting. As shown in Figure 4, a pair of varactor diodes (SMV2019 by Skyworks) is installed in a back-to-back configuration across the gap between the isolated circular sensing patch and the rest of the ground plane. Resistors (475 MΩ) are added in parallel with the varactor diodes to provide a conducting path for discharging low-frequency built-up charge across the varactor.

**Figure 5:**
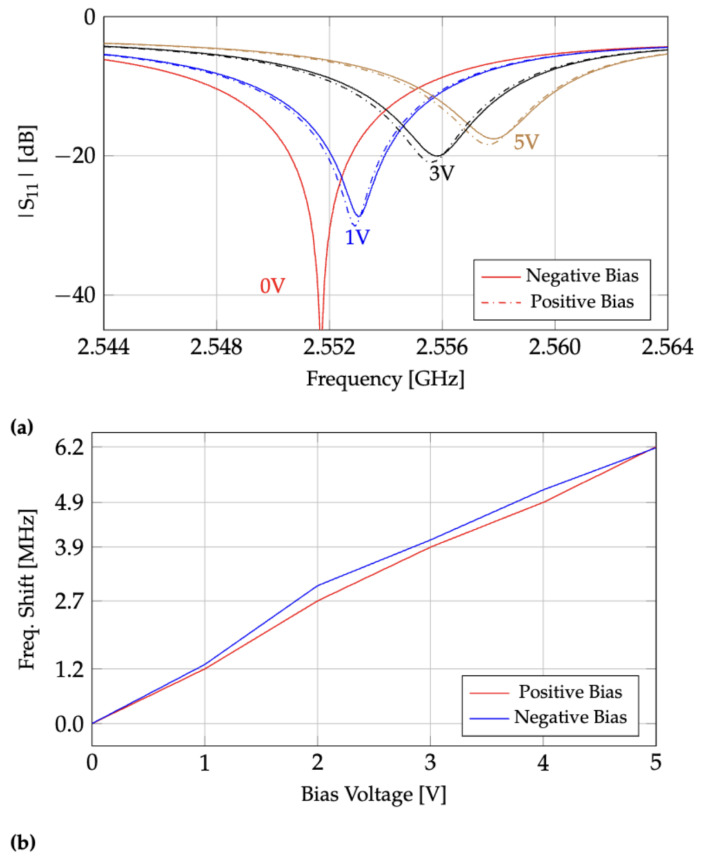
(**a**) Measured S_11_ parameter of the sensor for positive and negative bias voltages applied to the isolated sensing patch. Note that there is a total loss of 4.2 dB from the cables connected to the VNA ports. (**b**) Frequency shift versus the positive and negative bias voltages applied to the terminals of the back-to-back varactor diodes across the gap. This plot is extracted from Figure 5a. Note that the positive and negative biases are with respect to the sensor’s ground plane.

The addition of the electric field sensing patch, diodes, and resistors adds loss to the resonator, resulting in a reduction in the unloaded quality factor. The measured unloaded quality factor for our sensor is 900 as compared with 1340 for the same CLAF-SIW without the isolated patch sensing region [14]. A Keysight ENA E5063A vector network analyzer was used to measure the input reflection coefficient (S_11_ scattering parameter) at the antenna port of the fabricated resonator for different bias voltages directly applied to the gap of the sensing patch. Figure 5a shows measurements of the reflection coefficient for both positive and negative bias voltages. The external antenna is capacitively coupled to the cavity using a wire probe located at the center of the cavity where the resonant mode field is maximum [14]. The insertion length of the probe is adjusted such that critical coupling is achieved ($|S_{11}|$ ⟶ 0) when the applied reverse bias voltage is 0. A range of 0 to 5 V results in a variation of the resonant frequency of 2551.7 to 2557.9 MHz, respectively, providing a sensitivity of 1.24 MHz/V over a bandwidth of approximately 6.2 MHz. The capacitance of the varactor diode exhibits approximately linear behavior up to 5 V applied reverse bias voltage [13]. Accordingly, this can be observed in Figure 5b, where a linear model can fit the shift in resonant frequency. Both negative and positive bias voltages show a similar response. Note that a larger isolated sensing patch results in a larger resonant frequency shift; however, the quality factor is reduced. For instance, increasing the radius of the sensing patch from 5 mm to 7 mm will increase the variation in the resonant frequency, Δf from 6.2 to 28 MHz for a reverse bias voltage change from 0 to 5 V. This improves the sensitivity by a factor of 4.5. However, the increased resonant frequency sensitivity comes at the cost of a significant reduction in the unloaded quality factor from 900 to 100.

## 3. Measuring a Time-Varying Electric Field

### 3.1. Experimental Setup

**Figure 6:**
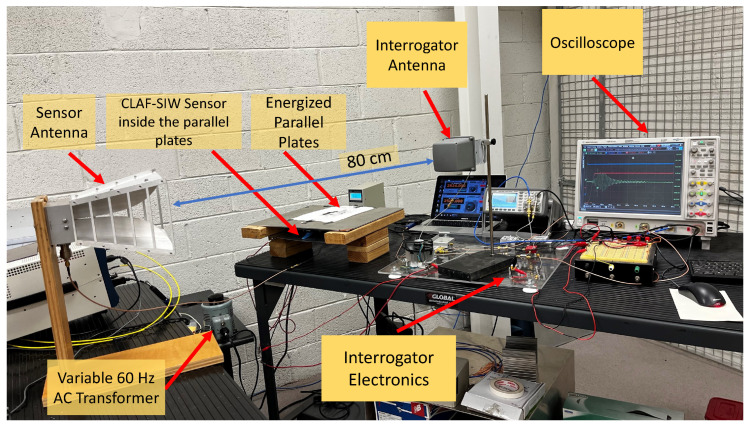
Photo of the electric field measurement setup. The CLAF-SIW resonator-based sensor is positioned between parallel plates, energized by a variable AC voltage. The two antennas of the sensor and the interrogation system are 80 cm apart.

Figure 6 shows the experimental setup used to test the performance of the developed wireless electric field sensor. The CLAF-SIW sensor is placed between two parallel conducting plates with a spacing of 4.5 cm. A variable sinusoidal voltage ranging from 0–140 V_rms_ is applied to the plates. For the proof-of-concept testing, an external horn antenna with a gain of 10 dBi is connected to the CLAF-SIW sensor using an SMA adaptor at the wire probe feed (the antenna would be ultimately integrated with the sensor resonator in a real application). An antenna (MD24-12 from Laird) with 12 dBi gain is connected to an interrogation system that transmits a 2540 MHz radio frequency (RF) signal to the CLAF-SIW resonator. As shown in Figure 6, the sensor and interrogator antennas are spaced 80 cm apart so that they operate in the far-field region.

**Figure 7:**
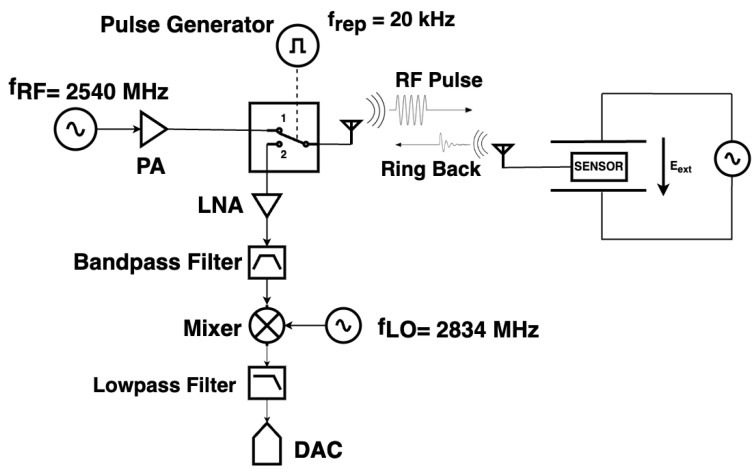
Block diagram of the ringback interrogation system and CLAF-SIW sensor placed between parallel plates where a known value of an external electric field is generated for testing the performance of the sensor.

Figure 7 shows the block diagram of the interrogation system. To energize the CLAF-SIW resonator, an RF source transmits a 2 dBm pulsed continuous wave (CW) signal at a near adjacent frequency to the resonant frequency of the sensor. This results in an effective isotropic radiated power (EIRP) of 32 dBm when the antenna gain is included. The power level of the RF pulse can have an impact on the sensitivity of the measurements. A higher power level improves the signal-to-noise ratio (SNR). This provides a larger amplitude for the backscattered signal so that we can derive the resonant frequency more accurately, resulting in a higher measurement sensitivity. The transmit–receive pulse cycle has a repetition frequency of 20 kHz with a 20% “ON” duty cycle. When the switch is in position 1, a CW pulse is transmitted to the sensor for 10 μs. This will energize the sensor to a near equilibrium state as the CLAF-SIW resonator has a loaded quality factor of 360 when connected to the antenna. The switch then moves to position 2 to receive the back-scattered ringback signal from the CLAF-SIW resonator for 40 μs. The output is amplified, filtered, and then downconverted using a mixer and a local oscillator at a frequency of 2834 MHz. A digital oscilloscope with segmented memory records multiple downconverted ringback signals.

**Figure 8:**
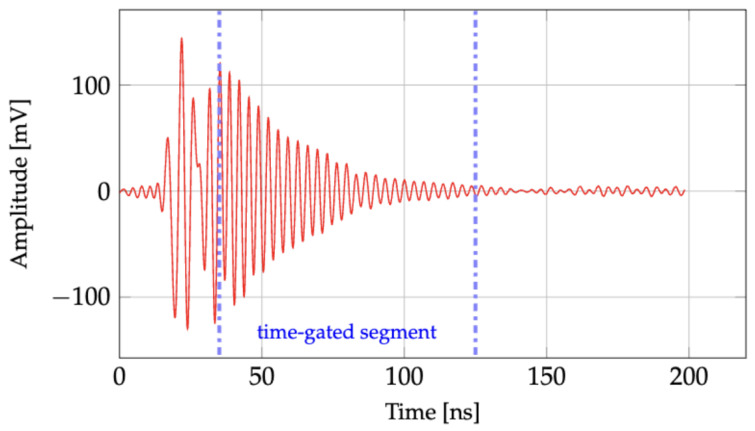
A sample of a measured downconverted ringback signal captured by the remote interrogation system shown in Figure 7. The time-gated segment primarily contains the antenna mode signal from the CLAF-SIW sensor.

To adequately capture an external sinusoidal electric field and its harmonics, approximately 15 to 30 samples/cycle are required. Figure 8 shows a sample of the downconverted ringback signal captured by the interrogation system. The ringback signal is first time-gated to remove reflections from the sensor’s antenna and the test environment. The antenna mode component of the signal (labeled as the time-gated segment in Figure 8) is then analyzed to determine the sensor’s resonant frequency. Referring to Figure 8, the ringback signal segment is received after a two-way delay of approximately 20 ns (5.4 ns for the 80 cm antenna–antenna air propagation path and 14.6 ns for the cables connecting the antennas to the sensor and the interrogator, as well as the interrogator inherent delays). Reflections from impedance mismatch at the transmit antenna and the structural mode of the receive antenna become apparent 5 ns after the RF source is disabled. Reflections from objects in the environment may be strong but typically decay rapidly as they are not high quality factor resonant structures. The feedthrough that shows up after approximately 100 ns in Figure 8 originates from the LO-IF isolation of the mixer and the RF switch. To obtain the sensor’s resonant frequency, 4 ringback signals are recorded and averaged, subjected to time gating, and then a Fast Fourier Transform (FFT) is applied. The resonant frequency of the sensor is then determined by calculating the frequency at which the absolute value of the Fast Fourier Transform (FFT) of the time-gated, averaged backscattered ringback signals is maximum. The resonant frequency calculation error is due to quantization error of the FFT analysis, random noise, or systematic error coming from multiple reflections, interference, and feedthrough of the interrogation system. To avoid systematic error, it is possible to calibrate the system for each specific environmental situation. Quantization error can be evaluated using the resolution of the FFT analysis, which in this work is Δf = 3.8 kHz. This value was determined by dividing the sampling frequency $f_{S}=1/\mathrm{time}\;\mathrm{step}=1/\left(62.5\;\mathrm{ps}\right)=16$ GHz by the number of points in each ringback signal $N_{S}\approx 4.2\;M$ points. Once connected to the antenna and placed between the parallel plates, the resonant frequency of the sensor varies from 2540.8 MHz to 2548.2 MHz. Using a local frequency (LO) of 2834 MHz for the mixer results in an intermediate frequency (IF) ranging from 285.8 MHz (period = 3.92 ns) to 293.2 MHz (period = 3.94 ns). Our sensor has a measured loaded quality factor of 360 (once connected to the antenna and the coaxial cable) which results in a ringback signal with a time constant of 45 ns. This provides sufficient time (approximately 10 periods) to adequately sample the received ringback signals.

### 3.2. Measurement Results and Sensor Response

**Figure 9:**
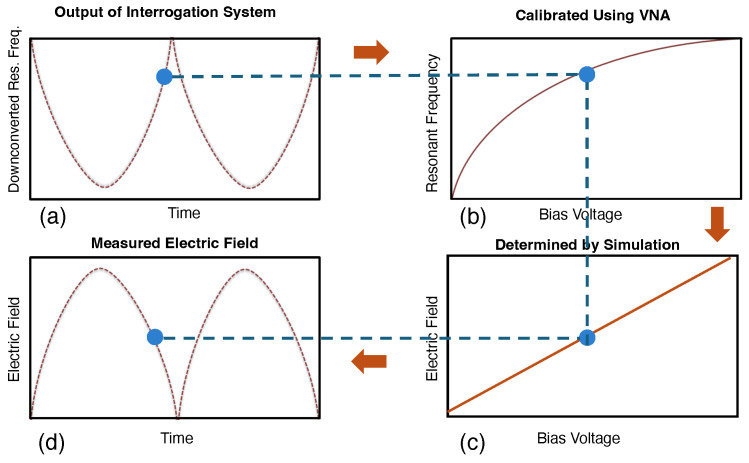
Illustration showing the procedure used to obtain the absolute value of the time-varying external electric field from the sensor’s measured time-varying downconverted resonant frequency: (**a**) the measured downconverted resonant frequency over time for a sinusoidal time-varying electric field, (**b**) relationship between the sensor resonant frequency and sensor patch gap bias voltage (derived from measuring the reflection coefficient of the sensor), (**c**) relationship between the electric field and the bias voltage (determined using a finite-element method solver), and (**d**) the final absolute value of the time-varying external electric field.

Figure 9 shows the procedure for determining the time-varying electric field based on the measured time-varying downconverted resonant frequency. The procedure involves mapping the variation in the sensor’s resonant frequency with time to the external electric field as

(1) $$\left|E_{\mathrm{ext}}\left(t\right)\right|=\chi \left(f_{\mathrm{res}}\left(t\right)-f_{o}\right),$$

where fres(t) is the measured downconverted resonant frequency that varies with the time-varying external electric field, $f_{o}$ is the downconverted resonant frequency when the applied external electric field is zero is constant for a given sensor under specific environmental conditions. Note that environmental conditions can cause a change in $f_{o}$. For example, the ambient temperature changes the size of the cavity under thermal expansion/contraction resulting in a change in the resonant frequency of the sensor even when the external electric field is zero. The function χ represents a mapping function that relates the measured variation of the resonant frequency with respect to $f_{o}$ to the absolute value of the applied external electric field. This function is determined through the calibration of the sensor.

Figure 9a shows an example of the measured resonant frequency for a sinusoidal time-varying electric field. Figure 9b shows the relationship between the measured resonant frequency and the bias voltage across the sensing patch gap, which was established by measuring the resonator’s reflection coefficient for different applied voltages (see Figure 5). A finite element method (FEM) electromagnetic solver is utilized to determine the relationship between the voltage induced across the gap of the sensor patch and an external electric field (see Figure 9c). The mapping from bias voltage to the external electric field depends on the size of the gap, the geometry of the sensor structure, and the angle of the sensor with respect to the external electric field. Hence, it needs to be calibrated accordingly. In our particular installation, the external electric field is perpendicular to the plane of the sensor to receive the maximum coupling between the external electric field and the sensor. As the orientation of the sensor changes with respect to the applied external electric field from the perpendicular position, the coupling between the external electric field and the sensor decreases, resulting in a drop in the induced voltage over the terminals of the varactor diodes. By establishing these relationships between measured resonant frequency, bias voltage, and external electric field, the function χ in (1) can be defined and this accurately determines the absolute value of the time-varying electric field waveform.

**Figure 10:**
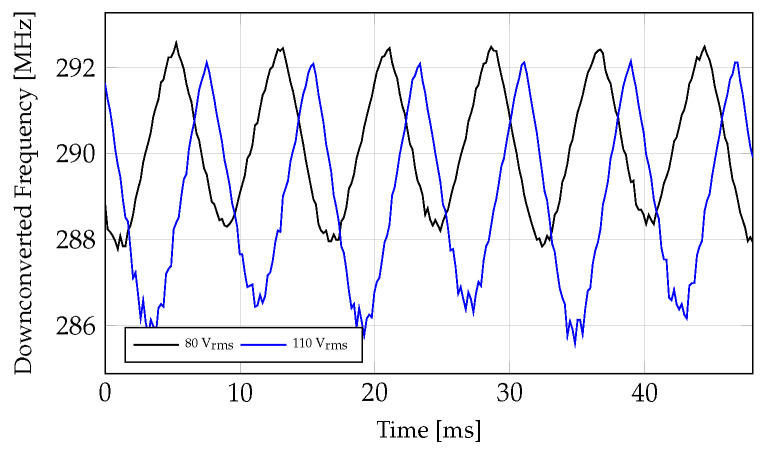
Measured downconverted resonant frequency versus time when 80 V_rms_ and 110 V_rms_ sinusoidal voltages are applied to the parallel plates of the test setup, resulting in electric fields of 2545 V/m and 3500 V/m, respectively. Note the downconverted frequency decreases for increasing the absolute value of the external electric field.

**Figure 11:**
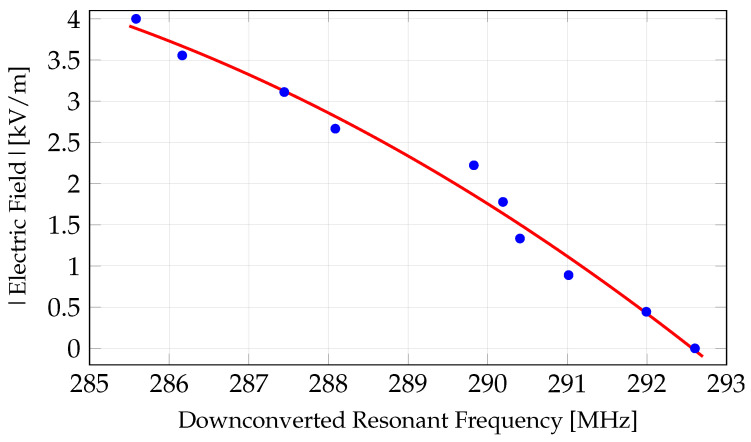
Measured absolute value of the external electric field as a function of the sensor’s downconverted resonant frequency using the ringback signal measurements as explained in Figure 9. The quadratic fitted curve is the function χ used in (1).

**Figure 12:**
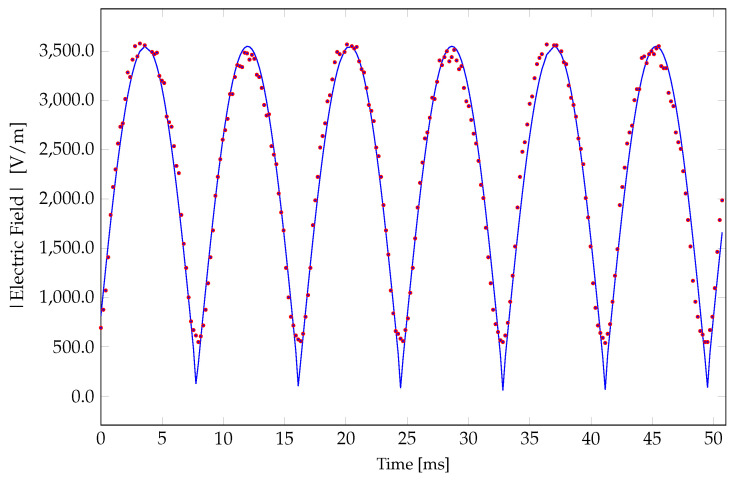
Measured electric field generated by applying a 60 Hz, 110 V_rms_ amplitude sinusoidal waveform to the parallel plate test system (data points with a 5 points/ms sampling rate). Comparison with the absolute value of a sinusoidal waveform of amplitude 3500 V/m (blue curve).

Figure 10 shows the measured sensor’s downconverted resonant frequency as a function of time for 60 Hz sinusoidal voltages of amplitude 80 and 110 V_rms_ applied to the parallel plates of the test setup. This corresponds to external electric fields of 2545 and 3500 V/m, respectively. Note, since $f_{\mathrm{LO}}$ >$f_{\mathrm{RF}}$, $f_{\mathrm{IF}}$ decreases for increasing $f_{\mathrm{RF}}$ and thus for increasing $\left|E_{\mathrm{ext}}\left(t\right)\right|$. The ripples around the minimum values, which correspond to the peak values of the applied external electric field waveform, occur due to a lower signal-to-noise (SNR) ratio. This is due to the roll-off of the C−V curve of the varactor diode for higher fields. Figure 11 shows the mapped absolute value of the external electric field as a function of downconverted resonant frequency obtained when a 60 Hz sinusoidal voltage with a range of amplitudes is applied to the parallel plates to generate a known external electric field. The fitted curve in Figure 11 provides the relationship between the external electric field and the downconverted resonant frequency, which is the function χ in (1). This mapping is valid for any electric field waveform. Figure 12 shows the reconstructed time-varying electric field for a 110 V_rms_ 60 Hz sinusoidal voltage applied to the parallel plates (generating an electric field of 3500 V/m). The absolute value of a 60 Hz sinusoidal waveform of amplitude 3500 V/m is also shown in Figure 12 for comparison. Differences between the reconstructed waveform and the expected waveform near the low field values are caused by the back-to-back varactor RC circuit configuration.

### 3.3. Performance Evaluation

The interrogation system transmits RF pulses to the resonator at 2540 MHz (see Figure 7). As shown in Figure 10, the downconverted resonant frequency of the resonator varies from 285.8 MHz to 293.2 MHz, depending on the applied external electric field. The measured downconverted ringback waveform, shown in Figure 8, is used to derive the resonant frequency. The time constant of the ringback signal in Figure 8 is approximately τ=45 ns. From the time-gated segment of the ringback signal, the loaded quality factor of the resonator, which includes the loading effect of the horn antenna and cable connected to the sensor, can be calculated as

(2) $$Q=\tau \pi f_{r}=\left(45\;\mathrm{ns}\right)\times \pi \times \left(2544.5\;\mathrm{MHz}\right)=360.$$

The maximum repetition frequency $f_{\mathrm{rep}}$ of the interrogator system (controlling the switch in Figure 7) is determined by the time $T_{c}$ needed to energize the resonator to at least 90% of its maximum [12] plus the time $T_{d}$ to allow the backscattered signal to decay to less that 1%. As such, $T_{c}=2.2\tau$ and $T_{d}=5\tau$. For τ=45 ns, the maximum repetition frequency is calculated as

(3) $$f_{\mathrm{rep}}=\frac{1}{T_{c}+T_{d}}=3.1\;\mathrm{MHz}.$$

In our system *n* consecutive measured downconverted resonant frequency values are averaged (i.e., *n* ringback signals analysed to produce one resonant frequency time sample) and *m* time samples are acquired for each period of the AC electric field. The highest frequency $f_{\max}$ of the external electric field that can be measured is then calculated by

(4) $$f_{\max}=\frac{f_{\mathrm{rep}}}{mn}.$$

For example, using n=4 and m=30 results in $f_{\max}\approx 25$ kHz.

**Figure 13:**
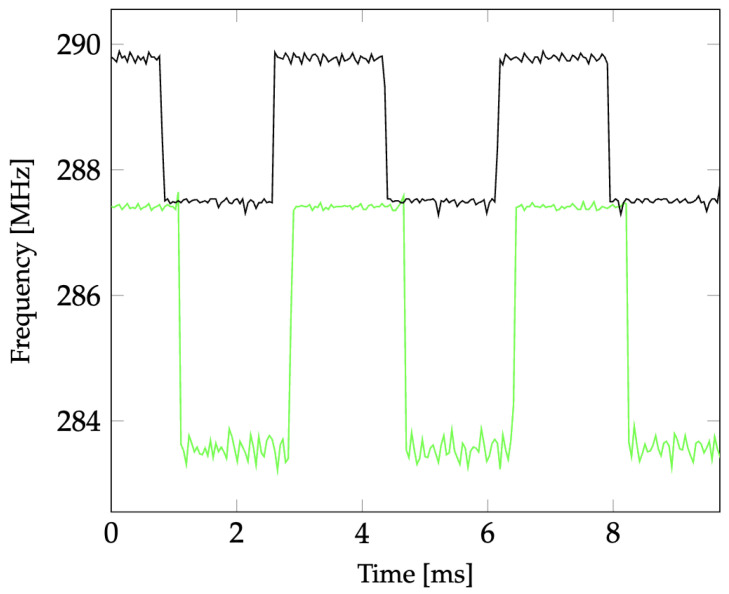
Measured downconverted resonant frequency versus time for applied 60 Hz square waveforms of 0 V to 2 V (black) and 2 V to 5 V (green) amplitude. The LO frequency of the downconverting mixer is 2834 MHz.

**Figure 14:**
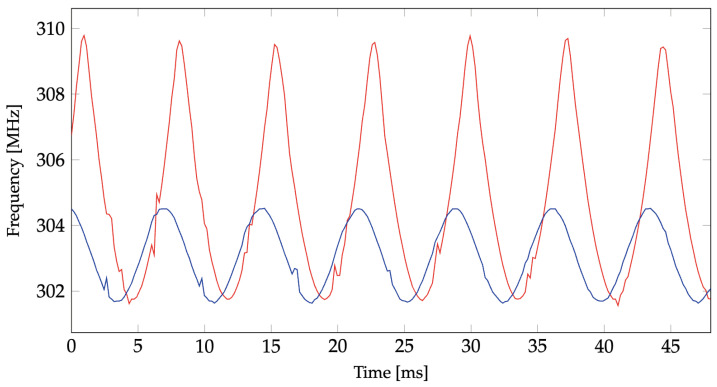
Measured resonant frequency over time for two 600 Hz sinusoidal waveforms. The peak values are 5 V (red) and 1.5 V (blue). The voltages are applied directly to the terminals of the varactor diodes. The LO frequency of downconverting mixer is 2854 MHz.

The performance of the sensor for different AC waveforms is analyzed by applying a voltage waveform to the terminals of the varactor diodes directly (i.e., wired connection). Figure 13 shows the variation of the downconverted resonant frequency of the sensor when a 60 Hz square wave of different amplitude values is applied to the varactor diodes. Figure 14 shows the measured downconverted resonant frequency over time for a 600 Hz applied sinusoidal waveform. This demonstrates that the sensor is capable of capturing frequencies higher than 60 Hz, which enables measuring the harmonics of a 60 Hz high-voltage system. In Figure 14, the downconverted resonant frequency varies from 309.9 MHz to 301.8 MHz when the applied voltage varies from 0 to 5 V. In Figure 13 and Figure 14, unlike Figure 12, the voltage is directly applied to the pair of back-to-back varactor diodes, and the zero-crossing of the reconstructed waveforms is not influenced by the characteristics of the back-to-back varactor RC circuit. Different LO frequencies are used to downconvert the ringback signals in Figure 13 and Figure 14, resulting in different IF frequencies. The frequency shifts are still in agreement with the mapping in Figure 11.

## 4. Conclusions

This paper presents the design, fabrication, and test of a chipless resonator-based CLAF-SIW sensor for measuring time-varying electric fields. This is especially useful in monitoring the electric field from high-voltage apparatus. The CLAF-SIW resonator employs a periodic electromagnetic bandgap structure, consisting of mushroom-cell patches around an air cavity to reduce dielectric loss and enhance the sensor’s quality factor. This increases the sensitivity of the sensor and decreases clutter interference. Furthermore, using the periodic EBG structure instead of conventional conducting vias allows for a contactless design, which addresses the manufacturing complexity of multilayer air-filled structures. A pulsed CW ringback wireless interrogation scheme is employed. RF pulses at a frequency near the resonant frequency of the resonator are transmitted and emitted ringback signals from the sensor are analyzed to detect changes in the resonant frequency of the sensor caused by the external electric field.

Table 2 summarizes the specifications of the proposed chipless CLAF-SIW electric field sensor. The design provides a planar platform with a conformal thin layer design allowing easy incorporation of a planar antenna to achieve a compact design. The sensor has an external electric field dynamic range of 6.9 kV/m. This corresponds to a maximum induced voltage of 5 V across the terminals of the varactor diodes, beyond which the varactor diodes capacitance becomes insensitive to the value of the reverse bias voltage. To increase the electric field dynamic range, a design modification, such as increasing the sensing patch size can be used. The maximum bandwidth of the external electric field is 25 kHz, whereas the minimum is limited by the time constant of the parallel resistor, varactor diode circuit.

In our demonstration of the sensor, a wideband horn antenna was used. However, a conformal antenna, such as a microstrip patch or a slot antenna can be directly integrated with the CLAF-SIW multilayer structure providing a thin, compact design. The proposed resonator design prototyped in this paper is fairly large, but can be made smaller using a higher operating frequency. Furthermore, a fast-sampling software-defined radio (SDR) platform can provide a more cost-effective alternative to the interrogation system. This change would reduce the size, weight, and cost of the interrogation system. The CLAF-SIW sensor also has the potential to be adapted for remote measurement of other measurands, such as temperature or strain, making it an adaptable and viable candidate for many sensing applications.

## Figures and Tables

**Table 1:** Design parameters of the CLAF-SIW resonator for electric field sensing.

Parameter	Label	Value (mm)
Width of Ground Plane	W_G_	147.2
Length of Ground Plane	L_G_	159.2
Width of Air Cavity	W_C_	73.3
Length of Air Cavity	L_C_	79.3
Sensing Patch Radius	R_SP_	5.0
Sensing Patch Gap	G_SP_	0.5
Mushroom Cell Length	L_M_	13.05
Mushroom Cell Width	W_M_	12.05
Mushroom Cell Via Radius	R_M_	5.0
Mushroom Cell-Cell Gap	G_M_	0.5

**Table 2:** Performance of the CLAF-SIW electric field sensor.

Parameter	Value
Sensor operating Frequency	2540.8 to 2548.2 (MHz)
Sensitivity to external electric field	1.86 (kHz)/(V/m)
Bandwidth of external E-Field	Low Frequency-25 kHz *
Dynamic Range of External E-field	up to 6.9 (kV/m)
Passive vs. Active	Passive
Demonstrated interrogation Distance	80 cm
Sensor Form Factor	Conformal Thin Layer

* Low frequency capability is limited by varactor and RC circuit discharge time.

## Data Availability

Data are contained within the article.

## References

[1] Huang Q.A., Dong L., Wang L.F. (2016). LC Passive Wireless Sensors Toward a Wireless Sensing Platform: Status, Prospects, and Challenges. J. Microelectromech. Syst..

[2] Islam M.M., Rasilainen K., Karki S.K., Viikari V. (2017). Designing a Passive Retrodirective Wireless Sensor. IEEE Antennas Wirel. Propag. Lett..

[3] Hallil H., Dejous C., Hage-Ali S., Elmazria O., Rossignol J., Stuerga D., Talbi A., Mazzamurro A., Joubert P.Y., Lefeuvre E. (2021). Passive Resonant Sensors: Trends and Future Prospects. IEEE Sens. J..

[4] Tan Q., Luo T., Wei T., Liu J., Lin L., Xiong J. (2017). A Wireless Passive Pressure and Temperature Sensor via a Dual LC Resonant Circuit in Harsh Environments. J. Microelectromech. Syst..

[5] Wen H., Chen C., Li S., Shi Y., Wang H., Guo W., Liu X. (2021). Array Integration and Far-Field Detection of Biocompatible Wireless LC Pressure Sensors. Small Methods.

[6] Amirkabiri A., Idoko D., Kordi B., Bridges G.E. High-Q Contactless Air-Filled Substrate-Integrated Waveguide (CLAF-SIW) Resonator for Wireless Sensing Applications. Proceedings of the 2021 IEEE MTT-S International Microwave Symposium (IMS).

[7] Bhadra S., Tan D.S.Y., Thomson D.J., Freund M.S., Bridges G.E. (2013). A Wireless Passive Sensor for Temperature Compensated Remote pH Monitoring. IEEE Sens. J..

[8] Athauda T., Chandra Karmakar N. (2019). Review of RFID-based sensing in monitoring physical stimuli in smart packaging for food-freshness applications. Wirel. Power Transf..

[9] Deng W., Wang L., Dong L., Huang Q. (2019). Symmetric LC Circuit Configurations for Passive Wireless Multifunctional Sensors. J. Microelectromech. Syst..

[10] Lin Y.C., Cai M.X., Yang Y.J. (2021). A wireless passive pressure sensor using microstructured ferromagnetic films with tunable effective permeability. J. Micromech. Microeng..

[11] Dey S., Bhattacharyya R., Sarma S.E., Karmakar N.C. (2021). A Novel “Smart Skin” Sensor for Chipless RFID-Based Structural Health Monitoring Applications. IEEE Internet Things J..

[12] Yazdani M., Thomson D.J., Kordi B. (2016). Passive Wireless Sensor for Measuring AC Electric Field in the Vicinity of High-Voltage Apparatus. IEEE Trans. Ind. Electron..

[13] Amirkabiri A., Bridges G.E., Kordi B. Resonator Substrate-Integrated Waveguide (SIW) Sensor for Measurement of AC Electric Fields. Proceedings of the 2018 International Symposium on Electromagnetic Compatibility (EMC EUROPE).

[14] Amirkabiri A., Idoko D., Bridges G.E., Kordi B. (2022). Contactless Air-Filled Substrate-Integrated Waveguide (CLAF-SIW) Resonator for Wireless Passive Temperature Sensing. IEEE Trans. Microw. Theory Tech..

[15] Zhang G., Tan Q., Lin B., Xiong J. (2019). A Novel Temperature and Pressure Measuring Scheme Based on LC Sensor for Ultra-High Temperature Environment. IEEE Access.

[16] Patre S.R. (2022). Passive Chipless RFID Sensors: Concept to Applications—A Review. IEEE J. Radio Freq. Identif..

[17] Brinker K.R., Zoughi R. (2022). A Review of Chipless RFID Measurement Methods, Response Detection Approaches, and Decoding Techniques. IEEE Open J. Instrum. Meas..

[18] Subrahmannian A., Behera S.K. (2022). Chipless RFID: A Unique Technology for Mankind. IEEE J. Radio Freq. Identif..

[19] Mayani M.G., Herraiz-Martínez F.J., Domingo J.M., Giannetti R. (2021). Resonator-Based Microwave Metamaterial Sensors for Instrumentation: Survey, Classification, and Performance Comparison. IEEE Trans. Instrum. Meas..

[20] Behera S.K., Karmakar N.C. (2020). Wearable Chipless Radio-Frequency Identification Tags for Biomedical Applications: A Review [Antenna Applications Corner]. IEEE Antennas Propag. Mag..

[21] Zhu L., Hà T.D., Chen Y.H., Huang H., Chen P.Y. (2022). A Passive Smart Face Mask for Wireless Cough Monitoring: A Harmonic Detection Scheme with Clutter Rejection. IEEE Trans. Biomed. Circuits Syst..

[22] Subrahmannian A., Behera S.K. (2022). Chipless RFID Sensors for IoT-Based Healthcare Applications: A Review of State of the Art. IEEE Trans. Instrum. Meas..

[23] Zemljaric B. (2011). Calculation of the Connected Magnetic and Electric Fields Around an Overhead-Line Tower for an Estimation of Their Influence on Maintenance Personnel. IEEE Trans. Power Deliv..

[24] Thomson D.J., Card D., Bridges G.E. (2009). RF Cavity Passive Wireless Sensors with Time-Domain Gating-Based Interrogation for SHM of Civil Structures. IEEE Sens. J..

[25] Jiang H., Chang Z.Y., Pertijs M.A.P. (2016). A 30 ppm < 80 nJ Ring-Down-Based Readout Circuit for Resonant Sensors. IEEE J. Solid-State Circuits.

[26] Ji Y., Tan Q., Wang H., Lv W., Dong H., Xiong J. (2019). A Novel Surface LC Wireless Passive Temperature Sensor Applied in Ultra-High Temperature Measurement. IEEE Sens. J..

[27] Yao J., Mbanya Tchafa F., Jain A., Tjuatja S., Huang H. (2016). Far-Field Interrogation of Microstrip Patch Antenna for Temperature Sensing without Electronics. IEEE Sens. J..

[28] Zhu L., Alkhaldi N., Kadry H.M., Liao S., Chen P.Y. (2018). A Compact Hybrid-Fed Microstrip Antenna for Harmonics-Based Radar and Sensor Systems. IEEE Antennas Wirel. Propag. Lett..

[29] Yao W., Lu H., Till M.J., Gao W., Liu Y. (2018). Synchronized Wireless Measurement of High-Voltage Power System Frequency Using Mobile Embedded Systems. IEEE Trans. Ind. Electron..

[30] Deslandes D., Wu K. (2003). Single-substrate integration technique of planar circuits and waveguide filters. IEEE Trans. Microw. Theory Tech..

[31] Ndoye M., Kerroum I., Deslandes D., Domingue F. (2017). Air-filled substrate integrated cavity resonator for humidity sensing. Sens. Actuators B Chem..

[32] Parment F., Ghiotto A., Vuong T., Duchamp J., Wu K. (2015). Air-Filled Substrate Integrated Waveguide for Low-Loss and High Power-Handling Millimeter-Wave Substrate Integrated Circuits. IEEE Trans. Microw. Theory Tech..

[33] Ghiotto A., Parment F., Martin T., Vuong T.P., Wu K. Air-filled substrate integrated waveguide—A flexible and low loss technological platform. Proceedings of the 2017 13th International Conference on Advanced Technologies, Systems and Services in Telecommunications (TELSIKS).

[34] Bayat-Makou N., Kishk A.A. (2018). Contactless Air-Filled Substrate Integrated Waveguide. IEEE Trans. Microw. Theory Tech..

[35] Zhao X.F., Deng J.Y., Yin J.Y., Sun D., Guo L.X., Ma X.H., Hao Y. (2021). Novel Suspended-Line Gap Waveguide Packaged With Stacked-Mushroom EBG Structures. IEEE Trans. Microw. Theory Tech..

[36] Zhao X., Yan Z., Fan F., Sun D., Wei B., Wang E., Zhu H., Dai Y., Liu X. (2024). Contactless Air-Filled Substrate Integrated Cavity-Backed Slot Antenna Array with Improved Printed Ridge Gap Waveguide Feeding Network. IEEE Trans. Antennas Propag..

[37] Segura-Gómez C., Pérez-Escribano M., Biedma-Pérez A., Palomares-Caballero Á., Padilla P. (2024). Contactless AF-SIW phase shifters based on periodic structures at mmWaves. AEU-Int. J. Electron. Commun..

[38] Liu C., Tong F. (2015). An SIW Resonator Sensor for Liquid Permittivity Measurements at C Band. IEEE Microw. Wirel. Compon. Lett..

[39] Amirkabiri A., Idoko D., Bridges G., Kordi B. Temperature Sensing Using Wireless Passive Contactless Air-Filled Substrate-Integrated Waveguide (CLAF-SIW). Proceedings of the 2021 IEEE 19th International Symposium on Antenna Technology and Applied Electromagnetics (ANTEM).

[40] Pozar D.M. (2005). Microwave Engineering.

